# Progress in Adenoviral Capsid-Display Vaccines

**DOI:** 10.3390/biomedicines6030081

**Published:** 2018-07-26

**Authors:** Marija Vujadinovic, Jort Vellinga

**Affiliations:** Janssen Infectious Diseases and Vaccines, Pharmaceutical Companies of Johnson and Johnson, 2301 CA Leiden, The Netherlands; jvelling@its.jnj.com

**Keywords:** adenovirus, vaccine, adenovirus vector, capsid, capsid-display, hexon, fiber, penton, pIX

## Abstract

Adenoviral vectored vaccines against infectious diseases are currently in clinical trials due to their capacity to induce potent antigen-specific B- and T-cell immune responses. Heterologous prime-boost vaccination with adenoviral vector and, for example, adjuvanted protein-based vaccines can further enhance antigen-specific immune responses. Although leading to potent immune responses, these heterologous prime-boost regimens may be complex and impact manufacturing costs limiting efficient implementation. Typically, adenoviral vectors are engineered to genetically encode a transgene in the E1 region and utilize the host cell machinery to express the encoded antigen and thereby induce immune responses. Similarly, adenoviral vectors can be engineered to display foreign immunogenic peptides on the capsid-surface by insertion of antigens in capsid proteins hexon, fiber and protein IX. The ability to use adenoviral vectors as antigen-display particles, with or without using the genetic vaccine function, greatly increases the versatility of the adenoviral vector for vaccine development. This review describes the application of adenoviral capsid antigen-display vaccine vectors by focusing on their distinct advantages and possible limitations in vaccine development.

## 1. Introduction

The hallmark of vaccine-mediated protection against infectious diseases is the induction of B- and T-cell immune responses [1]. Recombinant replication-incompetent adenoviral human (AdV) vaccine vectors are generally engineered to genetically encode a transgene in the E1 region and utilize the host cell machinery to express the transgene and to induce antigen-specific T-cell and B-cell responses [2,3,4,5,6]. AdVs broad application as vaccine vectors is greatly facilitated due to several valuable attributes: (i) They contain a well characterized ~36 kbp in size genome which can easily be engineered; (ii) they can be grown to high titers in a number of available complementing cell lines such as HEK293 [7], 911 [8] or PER.C6 cells [9,10], and (iii) they have a broad cell tropism which enables them to efficiently transduce a wide range of dividing and non-dividing cells [11]. Importantly, in the clinical evaluations, AdV vaccines have been shown to induce potent T-cell and B-cell responses against pathogens, for instance HIV and Ebola, with a good safety profile [12,13,14,15]. By contrast, protein-based vaccines such as virus-like particles (VLP), typically in combination with an adjuvant, are usually highly effective at inducing mainly B-cell responses [16,17,18,19,20]. For some complex pathogens, effective vaccine-mediated protection requires potent multifaceted B- and T-cell immune responses [21,22]. This can be achieved using a prime-boost vaccination approach with, for example, an AdV prime and adjuvanted recombinant protein boost, containing the same antigens [23,24,25,26]. 

While the AdV prime-protein boost offers the advantages of well-known vaccine platforms, it is limited by increased cost of goods and potentially complex vaccine regimens. Combining the benefits of AdV and protein vaccination in a single vaccine formulation could be an attractive alternative. 

**Figure 1:**
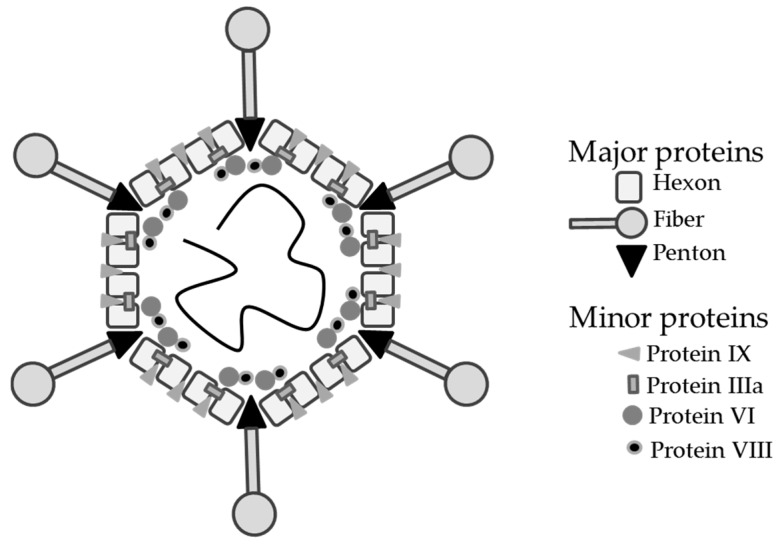
Adenoviral capsid. Schematic representation of an icosahedral AdV capsid organization with double-stranded DNA genome (black spiraling line). The outer capsid shell consists of three major capsid proteins hexon, fiber and penton base, and four minor capsid elements pIX, pIIIa and protein VI and VIII.

AdV vector capsid proteins can be engineered to display antigens on the capsid surface—referred to as capsid-antigen display vectors herein—thus offering the potential to combine genetic and protein-based vaccination in a single AdV vector. The AdV capsid consists of seven structural proteins; three major capsid proteins hexon, fiber and penton; and four minor ‘cement’ proteins protein IIIa (pIIIa), VI, VIII and protein IX (pIX) [27,28,29,30,31] (Figure 1), some of which have been modified to display heterologous peptide sequences for different purposes. 

Hexon, as a major capsid component, is a target for host immune responses against AdV [32], resulting in anti-vector immunity which may hamper with AdV vector efficacy [33,34,35,36]. To circumvent anti-vector immunity, hexon surface-exposed hypervariable regions (HVRs) can be replaced by alternative hexon sequences [32,37,38,39,40]. A human AdV5 (HAdV5) vector containing HAdV48 HVRs has been tested in phase I as an HIV vaccine [41]. Fiber and penton base are important for viral cell entry by binding to cellular receptor(s) (e.g., coxsackievirus and adenovirus receptor (*CAR*)) [42,43], and can be modified to alter AdV vector tropism to a specific cell type by replacing the native receptor binding motif by an alternative receptor motif sequence for gene therapy purposes [44,45,46]. Protein IX functions as a ‘cement’ protein and contributes to overall capsid stability [47,48]. Its surface-exposed C-terminus can accommodate fusion of peptides and proteins, for instance to alter the tropism or by generating viruses that express reporter proteins on the outer capsid surface for use in AdV trafficking studies [49,50,51,52]. Even though pIIIa allows small heterologous peptide/epitope fusions to its N-terminus, its tolerance for modification seems to be very low ([53,54], and M. Vujadinavic et al. [55]. 

**Figure 2:**
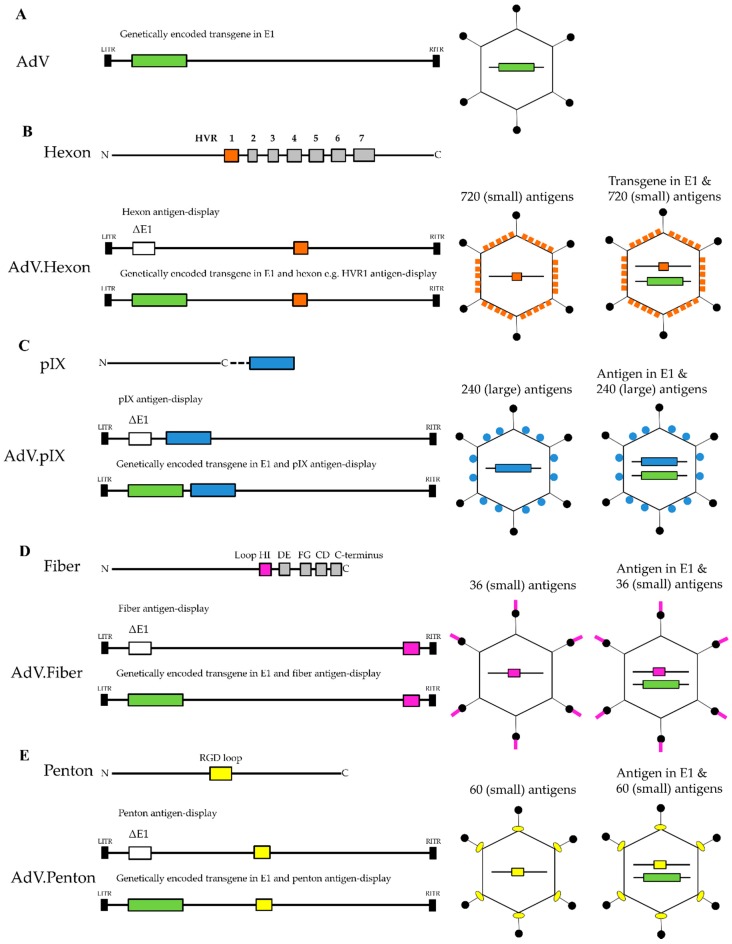
Schematic overview of capsid-display AdV vaccine vectors. (**A**) Replication-incompetent (ΔE1) AdV vectors encoding a transgene (green) in E1 (AdV.E1). (**B**) Hexon protein contains seven hyper variable regions (HVR1–7). The size in amino acids (aa) can vary per AdV type e.g., HAdV5 HVR1 44 aa (137–181), HVR2 6 aa (187–193), HVR3 7 aa (211–218), HVR4 13 aa (247–260), HVR5 15 aa (267–282), HVR6 11 aa (304–315) and HVR7 28 aa (421–449). Single epitope can be inserted in or substitute the native HVR sequence resulting in 720 copies per capsid. AdV. Hexon display vectors are generated (e.g., HVR1 orange) with or without a genetically encoded transgene in the E1 region (green). (**C**) pIX can display 240 copies of linear peptides or globular proteins (blue) by fusion of the antigen (blue) to the C-terminus (with or without a spacer, dotted line). AdV.pIX display vectors are generated with or without a genetically encoded transgene in E1 (green). (**D**) Epitopes can be inserted in fiber protein HI-, DE-, FG-, CD-, loops and C-terminus. For example, HI-loop (purple) AdV.Fiber display vectors contain 36 copies per capsid and can be generated with or without a genetically encoded transgene in E1 (green). (**E**) Penton Arg-Gly-Asp (RGD) loop (yellow) can be modified to present linear epitopes, resulting in 60 copies of epitopes per AdV capsid (AdV.Penton) with or without a without a genetically encoded transgene in E1 (green). Right and left Inverted terminal repeat (RITR-LITR).

In this review, we focus on studies describing capsid-antigen display AdV vectors with antigen peptide insertions in AdV hexon, fiber, penton and pIX (Figure 2 and Table 1), with the aim to establish their value as vaccine vectors by highlighting their distinct advantages and possible limitations. Such knowledge can contribute to the successful future application of AdV vectors as an antigen display platform. The availability of multiple modification sites in the AdV capsid allows the selection of the optimal AdV antigen-display strategy. These AdV capsid-display vectors induce antigen-specific immune responses without an adjuvant in animal models. Next to the VLP-like antigen-display, AdV capsid-display vectors can be engineered to genetically encode antigens allowing the benefits of AdV-based genetic and protein vaccination to be combined within one vaccine vehicle. 

## 2. Hexon Antigen Display

Hexon is an attractive choice for the generation of AdV antigen-display vectors because of the high number of surface-exposed antigen insertion sites (Figure 2B and Table 1). There are 720 hexon monomer proteins per capsid, which consist of a relatively conserved base and a top section containing HVR1–7 [28,29,82,83]. The size of each HVR loop differs per AdV type e.g., human adenovirus 5 (HAdV5) HVR1 44 amino acids (aa) [84]. HVRs are exposed on the outer capsid surface, however, their relative surface exposure can vary [84]. For instance, in HAdV5 the largest HVR1, HVR5 and HVR7 are located on top, while HVR2, HVR3, HVR4 and HVR6 are positioned lower towards the base [84]. Nonetheless, all HVRs contain AdV-specific epitopes and are at target for host antibody responses [32,84]. Consequently, pre-existing specific anti-AdV antibodies can neutralize the AdV vector and reduce vaccine immunogenicity [33,34,35,36]. To circumvent anti-vector immunity for gene delivery purposes, single (e.g., HVR1), or all (i.e., HVR1–7) HVRs, or the complete hexon have been replaced with their counterparts from alternative less-prevalent AdV [32,37,38,39,40,85]. Similar modifications have been made for the generation of antigen-display vectors by replacing some or all HVR sequences, or by inserting a target-specific epitope in the hexon HVR loop to display mostly linear epitopes on the outer capsid surface. 

Successful epitope-display and antigen-specific immune responses in mice have been demonstrated for HAdV5 expressing the target epitope on HVR1 [56,57,58,59,62,63] (*Aotus nancymaae* [58]), HVR5 [63,69,70] and HVR2 [70]. In particular, antigen-display via HVR1 is highly versatile. HVR1 was shown to accommodate linear epitope from different pathogens (e.g., HIV gp120) in a size range of 8 to 30 amino acids (aa) resulting in potent antigen-specific immune responses [56,57,58,59,62,63]. However, the display of a 10aa-HIV gp120-epitope alone in HVR1 [59] or HVR5 [86] was unsuccessful. Surrounding this epitope with ‘spacer’-sequences (~15 aa) resulted in antigen display, suggesting that additional spacing or peptide engineering may rescue epitope presentation. The latter observations demonstrate the importance of epitope design (e.g., size of the epitope and presence of additional spacers) in combination with the appropriate HVR for successful epitope display to induce potent immune responses. HVR1 and HVR5 appear to display similar sized epitopes (~30 aa) inducing comparable antigen-specific immune responses [63]. HVR5 can display linear epitopes from different pathogens (e.g., Anthrax) [63,69,70] in the size range of 12 to 66 aa with potent antigen-specific immune responses in mice. In contrast, HVR2 has not been extensively utilized for epitope display. Similar sized epitope display via HVR2 or HVR5 demonstrate HVR2 to be less permissive for larger peptide insertions, with the induction of lower antigen-specific immune responses compared to HVR5 [70]. 

To generate AdV display vectors presenting more than one antigen per capsid or more copies of the same epitope from a single AdV vector, multiple hexon HVRs can be modified. Antigen-specific immune responses were detected with display via HVR1 (HIV, 7 aa) combined with His-epitope in HVR5, but not HVR2 [61], supporting previous results of poor immunogenicity against epitopes displayed via HVR2 [70]. The suboptimal antigen display via HVR2 may be due to poor accessibility of the epitope, since HVR2 is located away from the top relative to HVR1 and HVR5 [84]. Comparable immune responses against both antigens displayed via HVR1 and HVR5 [61] illustrate the potential of displaying different epitopes from a single AdV vector to generate a multivalent vaccine. 

To combine the benefits of genetic and protein-based vaccination, hexon-display vectors can be engineered to genetically encode an antigen resulting in a (multivalent) display-expression. HAdV5 hexon display-expression vectors encoding and displaying antigens from pathogens such as *Pseudomonas aeruginosa* outer membrane protein F (OprF) epitope (HVR5, 14 aa) [68,71,72,87], elicited potent (protective) immune responses against the displayed epitope and the encoded transgene in mice. Epitope display via HVRs may be a means to reduce or interfere with anti-vector immunity in the host and enhance transgene-specific immune responses with display-expression vectors [68,86,87]. For example, HAdV5 HVR1 malaria circumsporozoite (CS) display vectors were largely unaffected by anti-HAdV5 neutralizing antibodies in vitro, supporting the notion that the majority of anti-HAdV5 antibodies after immunization are directed against HVR1 [86,87]. 

To fully evade anti-vector immunity, replacement of all HVRs is necessary [32]. As a result, use of HAdV5-based hexon-antigen display vectors remains restricted [88,89]. Vectors derived from rare AdV types such as HAdV26 [90] and chimpanzee AdV type 3 (CAdV3) [91] are less likely to be limited by pre-existing immunity in the human population. Due to variations in the HVR loop aa sizes and possibly surface-exposure among AdV types (e.g., HAdV5 HVR1 has 30 aa vs. HAdV48 HVR1 which has 8 aa), it is important to determine whether alternative AdV types can be successfully used to display antigens via different HVRs. Epitope display via hexon using alternative AdV types was demonstrated with HAdV3 [64,65], and simian adenovirus 25 (SAdV25) [66,67] vectors, with both vectors inducing potent immune responses in mice. For the HAdV3 vector, a 15 aa enterovirus epitope was successfully displayed via HVR1, HVR2 and HVR7, whereas display via HVR4 and HVR5 was unsuccessful [64,65]. Overall, these results suggest that alternative AdV types can be successfully used to display antigens via different HVRs to a similar extent as HAdV5 vectors. 

3. pIX-Display Vectors 

The most important advantage of pIX-display vectors is that unlike hexon, pIX tolerates fusion of larger peptides to its C-terminus (Figure 2C and Table 1). Protein IX minor capsid protein acts as a capsid stabilizing element in the AdV capsid [47,92]. It is believed to have additional functions as a transcriptional activator [93] and suppressor of the host-cell anti-viral response, thereby contributing to optimal virus production [94]. AdV vectors lacking pIX can be generated without deleterious effects on viral replication [95], albeit at a cost of reduced thermostability [96] and impaired packaging of full-length genomes [97]. There are 240 protein IX copies per capsid, arranged to protrude from the hexon cavities to expose the C-terminus tail to the outer surface [98,99] (Figure 1). Protein IX has proven suitable for the display of linear and globular functional proteins, for example, a polylysine motif or an eGFP protein, on the outer capsid surface [49,50,51]. This trait has been exploited for cell-specific AdV vector re-targeting and purification by fusion of specific ligands [51,100,101,102], or as a tool for AdV tracking in vitro and in vivo by fusion of functional reporter proteins [49,50,103]. Even though the pIX C-terminus is accessible on the outer capsid surface [104] it is embedded within the hexon cavity [98]. To increase the accessibility of the fusion peptide on the outer capsid surface, an alpha helical spacer can be added to lift the heterologous peptide fused to the pIX C-terminus out of the hexon grove [100]. 

In a HAdV5 vector, pIX was shown to display different antigens in a size range of 15 kDa to ~70 kDa (e.g., *Yersinia pestis* antigens) [73,74,76], inducing potent antigen-specific immune responses. Display of *Yersinia pestis* antigens via pIX elicited antigen-specific humoral responses and provided protection against lethal challenge in mice [73]. In addition, expression of pIX and the fused antigen in vivo is observed [105], which may contribute to potent immune responses against the pIX-fused antigen [74,76]. 

The potential of alternative AdV types for the generation of pIX-display vectors was confirmed by HAdV35 [77,78] and HAdV48 vectors [75], which induced potent antigen-specific immune responses in mice. HAdV35 displaying a ~15 kDa malaria CS antigen and encoding a CS transgene in E1 elicited higher immune responses than the transgene-only vector or CS protein alone. This effect could not be achieved by mixing the CS protein with the vector, suggesting that the antigen needs to be displayed on the outer capsid surface for induction of enhanced antigen-specific humoral responses [77]. HAdV35 vectors displaying HPV protein L2 epitope-repeats via pIX (e.g., HPV type 6, 31, 33, 16 (93 aa)) elicited L2-specific immune responses against the HVP types included and not included in the vaccine, demonstrating the potential of using pIX to display multiple linear epitopes in a repetitive confirmation from a single AdV vector to generate a multivalent AdV display vaccine [78]. In some instances, alternative AdV vectors were shown be more potent at inducing antigen-specific immune responses. For example, HAdV48 displaying 16 aa and 24 aa *Trypanosoma cruzi* epitopes outperformed the HAdV5 display vector by inducing higher B- and T-cell responses in mice [75]. This difference may be explained by a difference in pIX-antigen capsid incorporation efficiency between HAdV5 and HAdV48, resulting in the observed variation in potency. Previous studies in non-human primates also indicated that HAdV48 and HAdV5 induce differential innate responses which might result in differences in adaptive immune responses to the vector and the antigen [5]. 

## 4. Fiber- and Penton-Display Vectors

Fiber and penton proteins are appealing targets for epitope insertion because fiber trimers and penton pentamers are highly exposed on the 12 AdV icosahedral capsid vertices [106] (Figure 2D,E and Table 1). Fiber and penton are pivotal for cell entry [106,107]. Fiber initiates the interaction with the primary cellular receptor followed by secondary receptor interaction by penton, resulting in cell entry [42,108]. Modifications to these proteins may impact their binding efficiency to cellular receptors and consequently cell entry, which can be detrimental to vector potency and/or production in producer cell lines. Fiber consists of three distinct domains: (i) a C-terminal knob domain which binds to the primary receptor, (ii) the central shaft region consisting of flexible β-sheets, (iii) and a highly conserved N-terminal tail which interacts with the penton base [106,109]. Penton binding with secondary integrin receptors is mediated by the highly conserved Arg-Gly-Asp (RGD) motif which is embedded in the most variable region of the otherwise highly conserved penton base [106,110,111,112]. 

Successful full fiber substitutions, knob and shaft and shaft-only substitutions with counterpart alternative AdV fiber domains have been demonstrated, mainly for AdV vector retargeting in gene therapy [113,114,115,116,117], although the potential of AdV vector retargeting for vaccination purposes has been demonstrated. Chimera HAdV5 containing a HAdV35 species B fiber showed efficient transduction of antigen-presenting dendritic cells, suggesting potential for targeting specific cells in vivo [118,119]. In AdV-based HIV vaccine development, HAdV fiber chimera vectors induced potent (protective) immune responses in mice and monkeys [120,121,122]. Less invasive fiber modifications for AdV vector retargeting have been demonstrated. Several different receptor ligand peptides up to 55 aa in length were inserted into the surface-exposed fiber knob HI-, DE-, FG-, CD-loops and the C-terminus [81,123,124,125,126,127]. Similarly, to facilitate AdV binding to an alternative receptor penton base, modifications were achieved with replacement of the RGD motif for a different receptor binding motif [128,129]. 

Few studies demonstrate the potential of epitope-display via fiber [60,79,80,81] and only one via penton [79] (Figure 2D,E and Table 1). HAdV5 vectors displaying an influenza epitope via either the hexon HVR5, fiber HI-loop, penton RGD loop, or pIX, all induced antigen-specific immune responses in mice [79]. However, when mice were immunized with either the same AdV particle number or the same epitope copy number, influenza-specific humoral and cellular responses were highest with the fiber-display vector [79]. This observation is interesting considering that the fiber is the least abundant protein (36 copies vs. 60 of penton, 720 of hexon, and 240 of pIX) in the AdV capsid, suggesting that epitope accessibility and presentation is more important than the number of epitopes displayed per AdV capsid. HAdV5 vectors displaying a 14 aa *P. aeruginosa* OprF epitope via fiber HI-loop, FG-loop, CD-loop, DE-loop, C-terminus and hexon HVR5 induced potent immune responses in mice [81]. In contrast to immunization with HAdV5 OprF transgene vector, the fiber FG-loop display vector induced protection against *P. aeruginosa* even in the presence of high anti-HAdV5 pre-existing immunity [81], clearly demonstrating the benefits of AdV fiber-antigen display vectors. 

## 5. Concluding Remarks

AdV capsid-display vaccine vectors can be generated by inserting antigens or epitopes into hexon, fiber, penton and pIX. The tolerance for epitope insertion varies per capsid protein and seems to depend on the specific region that is targeted (e.g., hexon HVR1 vs. HVR2) and antigen properties (e.g., epitope size, conformation and biophysical properties). Hexon, fiber and penton proteins typically allow the insertion of small and linear T-cell or B-cell epitopes, and such limits to antigen size and confirmation might limit their utility for vaccine development. In contrast, by allowing fusion of both linear and larger globular antigens to its C-terminus, pIX has the potential to induce broader or stronger antigen-specific immune responses. The studies described in this review indicate a wide range of successful pIX-antigen fusions in terms of peptide size (i.e., 15 to ~70 kDa). However, there might be some peptide characteristic other than size, such as charge, which can influence successful pIX-display (e.g., pIX-HA) [130,131]. The pIX-antigen display as well as the expression of pIX-antigen contributes to the potency of these vectors, a characteristic which distinguishes the pIX-display platform from conventional VLP platforms. Since AdV vectors can be generated without pIX, suboptimal pIX-antigen capsid incorporation (e.g., 240 copies vs. 100 copies per capsid) may pose as a risk for consistent AdV manufacturing and/or vector quality. 

VLP particle-based epitope display has been explored in vaccine development using, for instance, adeno-associated virus (AAV) [132] or various other viruses [133,134,135,136]. AdV display-vectors have the potential to carry multiple antigens for instance in hexon (e.g., HVR1 and HVR5) or fiber (e.g., HI-loop and FG-loop), or epitope insertion in different capsid proteins (e.g., hexon and pIX). Insertion of multiple epitopes can be exploited to generate single AdV vectors displaying multiple epitopes, or greater numbers of the same epitope per capsid. In addition, AdV display vectors can be engineered to genetically encode an antigen to enhance antigen-specific humoral and cellular responses, combining the benefits of protein and AdV-based vaccines. 

Several strategies exist to overcome the impact of pre-existing immunity to highly prevalent vectors (e.g., HAdV5) on immunogenicity. For example, alternative AdV vectors (e.g., HAdV26 or ChAdV3) [90,91] with lower prevalence in human populations are available for genetic vaccination and/or capsid-display. Secondly, epitope insertions in the AdV capsid may reduce anti-AdV vector immunity [68,86,87]. Due to the spectrum of anti-AdV immune responses (e.g., anti-fiber and anti-hexon) in humans [137,138], it remains to be determined whether epitope display may indeed be sufficient to evade anti-AdV immunity in the clinic. 

One possible limitation of antigen-display on the AdV capsid may be that the neutralizing antibodies raised against the displayed epitope (e.g., HVR1-CS display) can neutralize the AdV capsid-display vector encoding a transgene, resulting in reduced (T-cell) immune responses against the encoded transgene after second administration [87]. The AdV capsid-display studies so far focus on homologous prime-boost regimens; to test the full potential of the platform it might be interesting to explore heterologous prime-boost regimens as well. However, in most homologous prime-boost regimens, AdV antigen-display vectors show potent (protective) antigen-specific immune responses against many different pathogens. The repetitive epitopes displayed on the AdV capsid enhances the induction of humoral immunity due to B-cell receptor crosslinking [139,140], which might be an advantage over genetic AdV vectors for the generation of antigen-specific nAb responses. Furthermore, the difference in responses of capsid-modified and genetic vectors might be attributed to differential processing of the antigen in hosts cell. 

To date, available publications offer little insight into the possible effects of the modification on the capsid protein’s additional biological function (e.g., AdV particle hexon-mediated transport to nucleus [141]), or critical AdV capsid-display particle characteristics such as viral titers yields, genetic stability in producer cell lines, particle stability, or appropriate formulation buffers. Yet, successful advancement of AdV capsid-display vectors depends highly on a potent vector generated at high yields in producer cell lines. Nonetheless, pre-clinical evaluations of the AdV capsid-display vectors describe potent (protective) antigen-specific immune responses against a range of complex infectious diseases, mostly without the addition of an adjuvant. Considering that the AdV capsid-display vectors are based on a well-established recombinant AdV vector platform, easily engineered and producible at high yields in producer cell lines, their suitability for commercial application is encouraging [10,142]. 

## Figures and Tables

**Table 1:** AdV-display vectors.

AdV Location	Vector	Disease and Antigen/Epitope (Size aa)	Other Modifications		Reference
			Transgene in E1	Capsid	
**Hexon HVR1**	HAdV5	Polio type 3 (8 aa)	No	No	[56]
		Malaria Circumsporozoite protein (CSP) (20, 24 aa) and (12 aa)	No	No	[57,58,59]
		HIVgp120 (10, 21, 24, 26 aa) and HIV gp41 and His6 (7, 6 aa)	Yes	Yes	[60,61]
		Chagas disease *Trypanosoma cruzi* gp83 (24 aa)	No	No	[62]
		Human papillomavirus (HPV) HPV16 L2 protein (29 aa)	No	Yes	[63]
	HAdV3	Enterovirus VP1 SP70 (15 aa) and Enterovirus VP1 SP70 and SP55 (15 aa)	Yes	Yes	[64,65]
	SAdV25 (AdC68)	Influenza A M2e and NP antigen (13 aa)	Yes	Yes	[66]
		Coxsackievirus and Enterovirus VP1 (6, 15 aa)	Yes	Yes	[67]
**Hexon HVR2**	HAdV5	HIV gp41 and Gag protein (39 aa)	Yes	No	[68]
**Hexon HVR5**	HAdV5	Anthrax *Bacillus anthracis* Protective antigen (PA) (16, 36, 66,143, 245 aa (eGFP))	No	No	[69]
		Model antigen RGD motif + His-linker (45, 55, 65, 75, 85, 95 aa)	Yes	Yes	[70]
		*Pseudomonas aeruginosa* outer membrane protein F (OprF) (14 aa)	Yes	Yes	[71,72]
**pIX C-terminus**	HAdV5	Plague *Yersinia pestis* (15, 37 aa)	Yes	No	[73]
		Ovalbumin (Ova) (43 aa)	No	Yes	[74]
	HAdV5 and HAdV48	Chagas-disease vaccine ASP2 and gp83 (26, 24 aa)	Yes	No	[75]
	HAdV5	HIV gp120 (67 aa)	No	Yes	[60]
		Friend Murine Leukemia Virus gp70, Gag and Ova (22, 43, 60, 70, 92) HAdV5/HAdV5.F35	Yes	Yes	[76]
	HAdV35	Malaria *P. falciparum Circumsprozoite protein (CSP)* (17, 20, 40 aa)	Yes	No	[77]
		Human papillomavirus (HPV) HPV6, 11, 16, 18, 31, 45, 52/58 L2 protein (93 aa)	No	No	[78]
**Fiber Knob-loop**	HAdV5	Influenza A HA (9 aa)	Yes	Yes	[79]
		Ovalbumin (20, 22 aa)	Yes	Yes	[80]
		*Pseudomonas aeruginosa* (14 aa)	Yes	Yes	[81]
